# The Neural Mechanism of Long-Term Motor Training Affecting Athletes’ Decision-Making Function: An Activation Likelihood Estimation Meta-Analysis

**DOI:** 10.3389/fnhum.2022.854692

**Published:** 2022-04-13

**Authors:** Ying Du, Lingxiao He, Yiyan Wang, Dengbin Liao

**Affiliations:** Department of Orthopedic, West China Hospital of Sichuan University, Chengdu, China

**Keywords:** decision-making, motor training, neuroimaging, brainmap, activation likelihood estimation (ALE), fMRI

## Abstract

Decision-making is an advanced cognitive function that promotes information processes in complex motor situations. In recent years, many neuroimaging studies have assessed the effects of long-term motor training on athletes’ brain activity while performing decision-making tasks, but the findings have been inconsistent and a large amount of data has not been quantitatively summarized until now. Therefore, this study aimed to identify the neural mechanism of long-term motor training affecting the decision-making function of athletes by using activation likelihood estimation (ALE) meta-analysis. Altogether, 10 studies were included and comprised a total of 350 people (168 motor experts and 182 novices, 411 activation foci). The ALE meta-analysis showed that more brain regions were activated for novices including the bilateral occipital lobe, left posterior cerebellar lobe, and left middle temporal gyrus (MTG) in decision-making tasks compared to motor experts. Our results possibly suggested the association between long-term motor training and neural efficiency in athletes, which provided a reference for further understanding the neural mechanisms of motor decision-making.

## Introduction

Decision-making refers to the advanced cognitive function to process information in complex situations (Johnson, 2006). The motor has the characteristics of behavior initiation, memory, and decision-making that make it easy to observe or measure the results of decisions, thus motor psychologists consider that the most suitable for decision-making research is the field of sports (Raab et al., 2019). Numerous studies have shown that long-term motor training can substantially change the neurological activation of the cerebral cortex (Kelly and Garavan, 2005; Leff et al., 2011; Morgan et al., 2015; Fernandes et al., 2017). Presently, motor-induced decreases in activity might occur in brain areas related to visual processing, such as the occipital pole (Duru and Balcioglu, 2018) and occipital fusiform gyrus (Herold et al., 2020). Motor psychologists used the “neural efficiency” hypothesis to explain the result (Babiloni et al., 2010; Li and Smith, 2021). Neural efficiency refers to the phenomenon in which task execution becomes an automatic and neural activity in specific brain regions decreases as skill levels increase (Neubauer and Fink, 2009; Karim et al., 2017). Meanwhile, long-term motor training promoted the formation of internal models in the brain that enabled athletes to perform tasks in a relatively stable and efficient manner (Imamizu et al., 2000). For example, a study on archers when performing aiming tasks found that experts invoked smaller and more focused neural networks, whereas novices activated a wide range of brain regions including superior frontal gyrus, inferior frontal gyrus, prefrontal lobe, primary motor cortex, superior parietal lobe and primary somatosensory cortex (Kim et al., 2014). In contrast, some authors have found that the ventromedial prefrontal cortex (Hiser and Koenigs, 2018), orbitofrontal cortex (Rudebeck and Rich, 2018), infralimbic cortex (Roughley and Killcross, 2021), and amygdala (Chang et al., 2015) were activated in experts during decision-making. To summarize, changes in cortical activation caused by extensive motor training might be to satisfy better decision-making.

An “expert-novice” paradigm has been developed in the field of motor cognition (Del Villar et al., 2007). An important approach to study the effect of motor training on cortical activation during executive decision-making was to recruit professionals with intensive motor experience (motor experts) as an experimental group and compare their brain activation with that of a control group (novices).

Currently, image fixation and eye-movement recording methods are mostly used to compare the decision-making function of experts and novices in different motor scenarios, yet these methods are likely to make the obtained data content inaccurate due to the subjectivity of the subjects (Brunyé and Gardony, 2017). The rapid development of neuroimaging techniques such as functional magnetic resonance imaging (fMRI) and positron emission tomography (PET) has informed further understanding of the neural mechanisms underlying the differences in decision-making behavior between experts and novices (Kable and Levy, 2015; Bertollo et al., 2020). However, these studies have reported inconsistent results, probably due to small samples or inconsistent analysis methods.

Several studies have found that in decision-making tasks, experts showed decreased activation in specific brain regions compared to novices (Del Percio et al., 2008; Babiloni et al., 2010; Blain et al., 2019). An fMRI study comparing the intensity of brain activation during the ball response task in table tennis players and non-athletes found that the bilateral middle frontal gyrus, right middle orbitofrontal area, left inferior temporal gyrus, left middle temporal gyrus, right angular gyrus, and bilateral lingual gyrus was significantly less activated in the players (Guo Z. et al., 2017) and similar results were found by another study (Kellar et al., 2018). These authors suggested that task-related brain networks were organized more centrally and efficiently as athletes improved their skills, so a possible reflected more efficient utilization of specific neural circuits or automation of task execution. A study investigated the brain activation of objects tracking decisions in basketball players and novices. The results showed that less cortical activation of the bilateral middle frontal gyrus, right middle orbitofrontal area, right paracentral lobe, right precuneus, left supramarginal gyrus, right angular gyrus, left inferior temporal gyrus, left middle temporal gyrus and bilateral lingual gyrus was observed in basketball players than in novices (Qiu et al., 2019). This might indicate that long-term motor training promoted higher decision-making efficiency and athletes did not need to use more information systems to make decisions from complex situations, which led to less activation of neural network areas associated with decision-making (Wang et al., 2017; Ludyga et al., 2020; Blazhenets et al., 2021).

However, two fMRI studies (Abernethy and Russell, 1987; Wong and Gauthier, 2010) showed that expert players were able to pick up more relevant information than novices in a selective decision-making task, electrophysiological data indicated more prefrontal positive activities in experts (Javier et al., 2014). Several recent studies have found that experts compared with novices could quickly perceive the actions of opponents and successfully respond, and the frontal areas were more activated, which might be related to the expert’s better ability in action planning and action understanding (Callan and Naito, 2014; Okazaki et al., 2015; Vernon et al., 2018). A randomized controlled trial recruited 15 basketball expert athletes and 15 novices to participate in an action decision-making task to analyze the correlation between gaze behavior and decision-making. The results showed that expert athletes had stable gaze fixation, accurate rate, and activation in the inferior parietal lobe and inferior frontal gyrus compared to novices (Wu et al., 2013). This suggested that experts might need more activation in the brain’s attention and sensorimotor networks to achieve better decision-making performance.

Taken together, the above evidence has suggested that long-term motor training could alter brain activation in decision-related areas, but the findings were inconsistent. On the other hand, in existing many imaging studies, there was a small sample size of subjects, and the selection of motor items, duration, and intensity of each motor also vary, which might lead to low statistical power and effect sizes and even inconsistent findings (Yarkoni, 2009). Therefore, to overcome the limitations of single studies and further elucidate the neural mechanisms underlying the effects of motor training on decision-making functions, an activation likelihood estimation (ALE) algorithm based on large amounts of data need to be introduced into the field of motor cognition (Yarkoni et al., 2011). The quantitative approach for ALE has been increasingly improved, obtaining rich information within the whole brain. Therefore, the current study applied the ALE method to compare brain activation differences between experts and novices during the execution of decision-related tasks. Our results provide a reference for further understanding the neural mechanisms of motor decision-making and promote the development of motor cognitive neuroscience.

According to the view of motor psychologists, athletes acquired motor skills through training rapid stimulus discrimination, decision-making, and specialized attention, but novices did not have. So they concluded that motor experts were better able to perform specific tasks with fewer neural resources, suggesting that long-term motor training could improve the neural efficiency of experts, reflecting the automation process of motor skills (Seiler, 2010; Denadai et al., 2017). Based on this view we predicted that experts might indicate an activity decrease in areas relevant to the motor decision process. The opposite hypothesis was that there was no significant difference in brain activation between experts and novices when performing decision-making tasks.

## Materials and Methods

### Literature Search

This ALE meta-analysis has been conducted following a strict protocol by using the Preferred Reporting Items for Systematic Reviews and Meta-Analyses (PRISMA) guidelines (Moher et al., 2009).

A literature search was conducted by PubMed,^1^ ISI Web of Science,^2^ Elsevier^3^ and Cochrane Library.^4^ All included articles were published in the English language until March 2021. The following search keywords were used: “athlete” OR “expert” OR “novice” OR “non-athlete,” “decision-making” OR “decision,” “functional magnetic resonance imaging” OR “fMRI” OR “positron emission computed tomography” OR “PET.” The retrieval formula was (“athlete” [Mesh Terms] OR expert OR novice OR non-athlete) AND (“decision-making” [Mesh Terms] OR decision) AND (“functional magnetic resonance imaging” [Mesh Terms] OR “fMRI” [Mesh Terms] OR “positron emission computed tomography” [Mesh Terms] OR PET).

The titles and abstracts were independently screened by two trained reviewers (YD and LXH). Two independent reviewers completed the entire article inclusion process, and only articles that both reviewers reached an agreement on were finally included. When two independent reviewers disagreed on whether to include articles, resolved the differences through discussion, and seed the assistance of a third reviewer (DBL) if they still could not be resolved.

### Inclusion and Exclusion Criteria

We selected studies considering the following inclusion criteria: (1) the samples included a group motor expert and a group novice; (2) motor experts with extensive experience were awarded the title of experts by their country, region, or school; (3) subjects performed motor decision-making task stimulus; (4) study with fMRI or PET; (5) the peak coordinates of brain activation areas were explicitly reported of motor experts and novices; (6) the reported results of activated foci were normalized to the Montreal Neurological Institute (MNI) (Collins et al., 1994) or the Talairach standardized stereotactic spaces (Paus et al., 1996); (7) whole-brain voxel analysis or at least one whole-brain analysis was performed; (8) subjects were healthy; (9) subjects were adults; (10) the language of studies was limited to English; (11) original research articles.

Exclusion criteria: (1) report coordinates were incomplete, or complete results have not been obtained after contacting the corresponding author; (2) research published in the form of conference reports, abstracts, etc.

**FIGURE 1:**
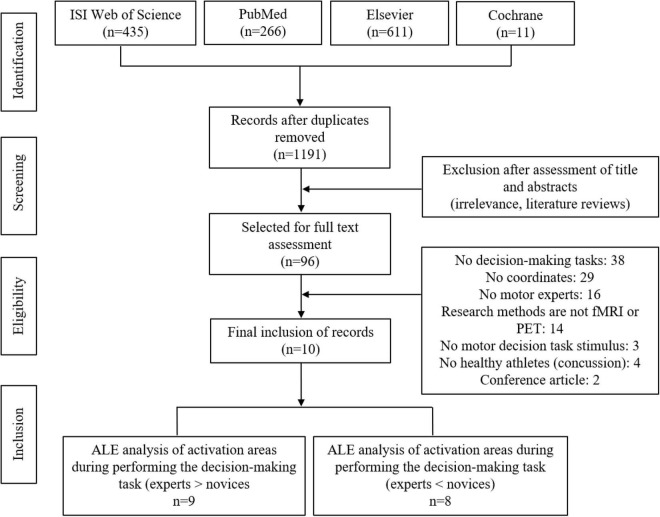
Flowchart describing the process of study selection.

These criteria identified 10 studies including 350 people (168 experts and 182 novices, 411 activation foci). Figure 1 shows the outcomes of the search process.

### Data Extraction

The two reviewers (YD and LXH) extracted the following information from each included study: authors, age, year of publication, study name, type of coordinates, brain analysis method, number of participants, the ratio of male to female participants, comparison conditions, handedness, Foci.

### Activation Likelihood Estimation Meta-Analysis

ALE is a quantitative meta-analysis method that uses a spatial variance model (3D Gaussian) to calculate the likelihood of each voxel being activated under a certain condition, thus obtaining the consistency of brain activation across multiple experiments (Laird et al., 2005; Eickhoff et al., 2009). Data processing was performed using GingerALE software (version 3.0.2)^5^ (Eickhoff et al., 2012). The difference in coordinate space (MNI vs. Talairach space) could be explained by converting the coordinates in Talairach to MNI space using icbm2tal in GingerALE (Lancaster et al., 2007). Finally, all activation coordinates were displayed in MNI space. We adopted a threshold for the map of the final ALE score graph with a familywise error (FWE) at *p* < 0.05 and a minimum cluster size of *k* > 10 mm^3^. For visualization, the ALE whole-brain maps were imported into Mango software (version 4.0.1)^6^ overly on a standardized anatomical MNI template (Colin27_T1_seg_MNI) (Dehghan et al., 2016).

## Results

### Study Selection and Characteristics

A total of 1,323 records were initially retrieved, and only 1,191 studies remained after deleting duplicates (*n* = 132). We screened potentially relevant articles by applying the inclusion and exclusion criteria (see Supplementary Material 1). Finally, 10 articles focusing on the brain activation of experts and novices while performing decision-making tasks were included in the present study. All included articles tested decisions made by experts and novices under the same stimuli. Among those papers, six (Wright et al., 2011; Wu et al., 2013; Balser et al., 2014; Wimshurst et al., 2016; Qiu et al., 2019; Blazhenets et al., 2021) determined the accuracy of the ball’s flight direction, three (Bishop et al., 2013; Wright et al., 2013; Xu et al., 2016) tested to determine the accuracy of opponent behavior, one (Guo-Zheng, 2016) tested response accuracy rate of ball block. Regarding the types of sports including basketball, tennis, hockey, volleyball, handball, badminton, soccer, etc. Table 1 described the detailed information of the included study.

**TABLE 1:** Basic information included literature.

References	Sample size	Imaging method	Expertise	Gender M/F	Space	Conditions	Handedness	Foci
Wu et al. (2013)	30	fMRI	Basketball	30/0	Talairach	Free-throw direction decision accuracy	Right	55
Balser et al. (2014)	32	fMRI	Tennis	16/16	MNI	Tennis flight direction decision	Right	22
Wimshurst et al. (2016)	30	fMRI	Hockey	19/11	MNI	Determine the accuracy of hockey hitting direction	/	76
Guo-Zheng (2016)	40	fMRI	Volleyball	20/20	MNI	The response accuracy rate of ball block	Right	38
Qiu et al. (2019)	47	fMRI	Basketball	47/0	MNI	Determines the direction and strength of the ball	Right	5
Blazhenets et al. (2021)	48	PET	Handball	48/0	MNI	Free-throw decision	Right	21
Xu et al. (2016)	34	fMRI	Badminton	19/15	MNI	Determines the direction and gender of the players	Right	24
Wright et al. (2011)	16	fMRI	Badminton	16/0	MNI	Decide where to drop the badminton	Right	50
Wright et al. (2013)	34	fMRI	Soccer	34/0	MNI	Decide the move direction in the opponent	Right	110
Bishop et al. (2013)	39	fMRI	Soccer	39/0	MNI	Decide an oncoming opponent’s movements	Right	10

### Single Dataset Activation Likelihood Estimation Analysis Results

Experts had 93 foci in 5 different experiments, the 3 regions activated included the right inferior temporal gyrus (ITG) (BA 37), right sub-gyral (BA 37), right middle occipital gyrus (MOG) (BA 19).

**FIGURE 2:**
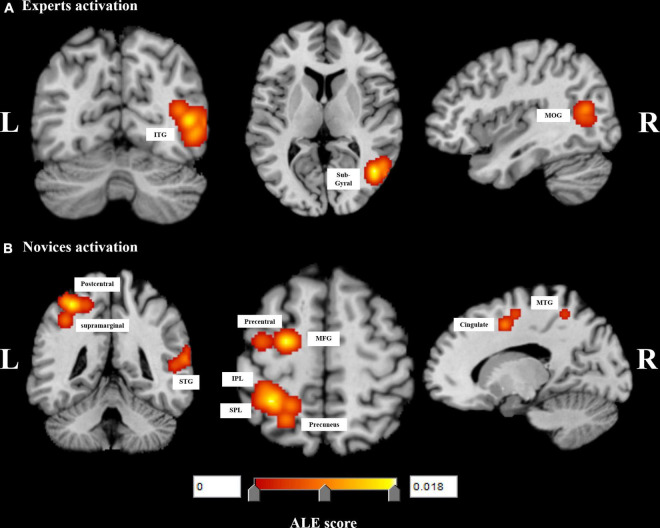
Significant meta-analysis results for **(A)** experts and **(B)** novices performing decision-making tasks. L, left, R, right, inferior temporal gyrus, ITG, middle occipital gyrus, MOG, superior parietal lobule, SPL, inferior parietal lobule, IPL, middle frontal gyrus, MFG, middle temporal gyrus, MTG.

Whereas novices had 124 foci in 7 different experiments. After completing the single data ALE analysis, novices activated the left precuneus (BA 7), left superior parietal lobule (SPL) (BA 7), left inferior parietal lobule (IPL) (BA 40), left middle frontal gyrus (MFG) (BA 6), left precentral gyrus (BA 4, BA 6), left cingulate gyrus (BA 24), left postcentral gyrus (BA 2), left supramarginal gyrus (BA 40), right middle temporal gyrus (MTG) (BA 37, BA 39), right superior temporal gyrus (STG) (BA22). Table 2 and Figure 2 showed the results of the single-dataset ALE analysis.

**TABLE 2:** ALE meta-analysis results according to experts, novices, and contrasts.

Anatomical region	Cluster	BA	MNI coordinates			Volume	ALE value
			X	Y	Z	(mm^3^)	(× 10^3^)
**Experts**							
R ITG	1	37	50	–68	–2	8,976	12.03
		37	56	–68	4		9.23
		37	62	–58	–4		8.64
R sub-gyral		37	46	–64	6		18.37
R MOG		19	40	–66	12		10.11
**Novices**							
L Precuneus	1	7	–24	–58	55	7,744	9.06
		7	–22	–48	56		8.86
L SPL		7	–34	–46	56		17.97
L IPL		40	–46	–34	40		8.81
L supramarginal gyrus		40	–40	–42	42		8.56
L MFG	2	6	–24	–6	54	7,120	17.73
		6	–28	–4	46		10.09
L precentral gyrus		4	–34	–12	64		9.07
		4	–34	–20	68		8.79
		6	–38	–6	52		8.65
L cingulate gyrus		24	–16	2	46		9.28
L postcentral gyrus		2	–44	–28	64		7.72
R MTG	3	37	50	–62	8	6,504	14.29
		39	46	–60	12		14.27
R STG		22	46	–52	20		9.15
		22	54	–48	8		8.97
		22	62	–46	12		8.84
		22	66	–42	18		8.83
***Experts* > *Novices***							
–	–	–	–	–	–	–	–
***Experts* < *Novices***							
L MOG	1	18	–20	–90	–4	27,000	17.46
		18	–14	–96	18		10.70
		19	–44	–80	18		9.18
		19	–48	–74	10		9.04
		18	–30	–84	6		7.95
		18	–26	–86	14		7.20
		19	–40	–88	12		5.06
L MTG		19	–44	–80	24		9.14
		19	–48	–62	16		8.83
		39	–40	–58	8		8.72
L IOG		17	–16	–98	–8		10.36
L lingual gyrus		18	–16	–104	0		8.62
L posterior lobe			–26	–82	–16		9.61
L precuneus		31	–30	–72	24		7.90
R lingual gyrus	2	18	30	–98	0	11,240	10.32
		18	20	–96	–10		10.05
		18	24	–86	–6		9.70
		18	10	–88	2		7.22
R MOG		18	28	–94	6		10.05
		18	22	–88	–2		9.92
R fusiform gyrus		19	30	–88	–10		9.64
R IOG		17	26	–100	–4		8.79

*ALE maps were computed at a familywise error (FWE) corrected threshold of p < 0.05, with a minimum cluster size of k > 10 mm^3^. BA, Brodmann area, L, left, R, right, superior parietal lobule, SPL, inferior parietal lobule, IPL, middle frontal gyrus, MFG, middle temporal gyrus, MTG, superior temporal gyrus, STG, inferior temporal gyrus, ITG, middle occipital gyrus, MOG, inferior occipital gyrus, IOG.*

### Dual Dataset Comparison Activation Likelihood Estimation Analysis

**FIGURE 3:**
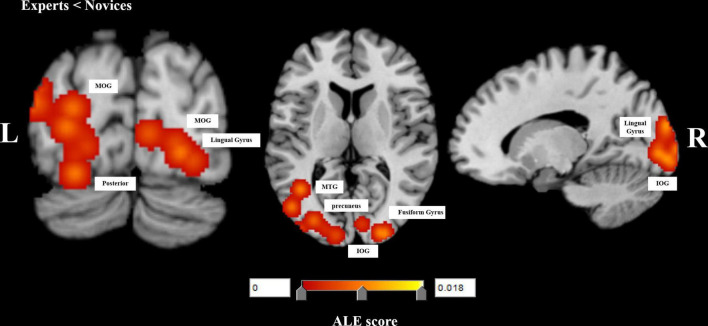
Significant meta-analysis results for comparison between experts and novices performing decision-making tasks. L, left, R, right, middle temporal gyrus, MTG, middle occipital gyrus, MOG, inferior occipital gyrus, IOG.

Experts did not have significant activation compared to novices. In contrast, novices were activated in the left middle temporal gyrus (MTG) (BA 19, BA 39), bilateral MOG (BA 18, BA 19), left posterior lobe, right lingual gyrus (BA 18), left precuneus (BA 31), right fusiform gyrus (BA 19), bilateral inferior occipital gyrus (IOG) (BA 17). Table 2 and Figure 3 showed the results of the ALE analysis for experts and novices comparisons.

## Discussion

### The Effect of Handedness on the Results of Activation Likelihood Estimation Analysis

Handedness might affect an individual’s skills and athletic performance (Raymond et al., 1996). Some studies also have found that handedness was associated with cognitive function (Buckingham et al., 2011; Somers et al., 2015). The results of an EEG study found that left-handed athletes had greater P300 wave amplitude than right-handed athletes, suggesting that left-handed athletes had an advantage in cognitive perception and observation, but slower processing than the right-handed athletes (Nakamoto and Mori, 2008). Almost all of the subjects in the existing fMRI literature of motor decision-making were right-handed since there were fewer left-handed athletes. Among the 10 articles included in this study, only one article did not report whether the subjects were right-handed or not (Wimshurst et al., 2016), and the remaining nine articles reported that the subjects were right-handed. Therefore, we considered the articles included in this study to be general.

### Summary of Activation Likelihood Estimation Meta-Analysis Results

Through systematic review and ALE meta-analysis of the included papers investigating brain activation in decision-making tasks by experts and novices, we found that experts activated the right ITG, right sub-gyral, and right MOG, but novices activated more brain regions including the left middle temporal gyrus (MTG) (BA 19, BA 39), bilateral MOG (BA 18, BA 19), left posterior lobe, right lingual gyrus (BA 18), left supramarginal gyrus (BA 40), right fusiform gyrus (BA 19), bilateral inferior occipital gyrus (IOG) (BA 17). Results of this ALE meta-analysis showed significant clusters in novices, and they were located in the bilateral occipital lobe (including the MTG, MOG, IOG, lingual gyrus, fusiform gyrus, and precuneus), left posterior cerebellar lobe, and left MTG compared to experts. This also confirmed that long-term motor training could improve the neural efficiency of motor experts, reflecting the automated process of motor skills.

### Brain Activation Contrasts Between Motor Experts and Novices in Decision-Making Tasks

There was substantial evidence that the occipital lobe played an important role in the processing of visuospatial information (Todorov and Sousa, 2017; Buening and Brown, 2018). Motor cognitive psychologists argued that decision-making was based on information, of which visual information was particularly important (Darren et al., 2016). The results of the present study showed that the occipital lobe had in novices compared to experts, suggesting that the brain required more visual information processing when performing decision-making tasks. The results also corroborated previous research (Jie, 2014; Filgueiras et al., 2017; Guo Z. et al., 2017; Blazhenets et al., 2021). When the brain was stimulated by complex motor scenarios, motor experts first transferred the stimulus information into the brain’s perceptual system. The brain recognized the information and matched it with tactical information extracted from long-term memory and then made a final decision based on the learned motor skills (Smits et al., 2014). In contrast, novices performed the same task needing to find usable information and invalid information, which led to more visual information systems being activated to process and analyze information. Therefore, we believed that the primary mechanism for decreased activation in the expert’s occipital lobe was that experts had higher neural efficiency, which implied task-specific brain function plus sparing (Neubauer and Fink, 2009; Ludyga et al., 2016). This suggested that visual information processing played a key role in motor decision-making behavior.

Compared with experts, the results of ALE analysis showed that novices activated the left posterior cerebellar lobe during the decision-making process. The cerebellar was more involved in motor regulating and motor learning (Ashida et al., 2019; Schmahmann et al., 2019). Several studies have indicated that the activation of the cerebellum during performing decision-making tasks could enhance decision-making planning, initiation, and control (Yarrow et al., 2009; Nowrangi et al., 2014; Kim et al., 2015). Previous studies have found that long-term professional motor training could alter cerebellar activation. For example, a study comparing brain activation in elite archers and non-archers during archery found that non-archers had more cerebellar activation than elite archers (Chang et al., 2011), which was consistent with the findings of Guo Z. et al. (2017). Authors indicated that experts through years of sports skill learning and training might develop precise and professional sports skills, which included the ability to rapidly regulate motor information and motor learning, whereas novices did not have the ability. Imamizu et al. (2000) suggested that motor could be accurately controlled by using internal models of the body and that after the cerebellum acquired internal models through long-term motor training, athletes were able to perform tasks in a relatively automated, energy-conserving processing mode. Based on this view we believed that the reduced activation of the expert cerebellum was due to the formation of internal models of the cerebellum.

The MTG (BA 39) was already known to be significantly involved in cognitive functions such as motor planning and information processing (Guo L. et al., 2017; Xu et al., 2019). The results of this study found that the left MTG was activated when novices made decision-making in sports, which reflected the fact that experts did not need to activate more cognitive areas of the brain. Motor psychologists have suggested that cognitive mechanisms might be the main reason for the different decision-making levels between experts and novices (Dew et al., 2009). Experts with extensive motor experience could facilitate sports memory and attention, especially the ability to increase the depth of attention and reduce the waning of attentional information in complex motor scenarios. However, when novices made motor decisions in face of unfamiliar motor conditions, activation of the left MTG might be due to further processing of motor behavior information which reconfirmed the neural areas involved in motor information processing (Woods et al., 2014). Therefore, we argued that long-term motor training in athletes led to the more efficient cognitive neural network to adapt to the demands of high intensity and high correctness, which induced the brain to automatically process information. This was consistent with previous research findings (Babiloni et al., 2010; Zhang et al., 2019; Dobersek and Husselman, 2021).

### Limitations and Future Research Directions

Potential limitations in this study should be mentioned. ALE analysis inevitably ignored the variation in each study and belonged to the statistical inference of fixed effects. Unlike a meta-analysis with complete activation maps, the data used in ALE were based on the reported data peak activation coordinates (Zhang et al., 2016). Therefore, it was unable to take into account studies without any significant categorical reporting, which might lead to systematic overestimation of biased results. Another limitation of this article was the gap between the designed motor situation and the real situation in included studies, which might make the findings not necessarily a true reflection of the actual movement, but rather an exploration of brain activation for decision-making tasks in a laboratory setting. Further development of realistic experimental designs or fMRI techniques could facilitate the direct exploration of decision-making in real motor situations, which was a direction of future research. Last, it was difficult to assess whether these activation regions were related to sports types due to the lack of comparisons between different motor experts, so further imaging studies were needed to compare brain activation alterations of different professional motor experts.

## Conclusion

This study took a cognitive neuroscience perspective to reveal differences in the neural mechanisms underlying the motor decision-making processes of experts and novices. Our study provided new and meaningful evidence that greater activation for novices compared to experts in the bilateral occipital lobe, left posterior cerebellar lobe, and left MTG, but a decreased activation was not detected.

## Data Availability Statement

The original contributions presented in the study are included in the article/Supplementary Material, further inquiries can be directed to the corresponding author/s.

## Author Contributions

DL was responsible for the conception and design of the study, explained the data results, and critically revised the manuscript. YD and LH performed the data acquisition and statistical analysis. YW completed the document screening and literature search. YD extracted the data and edited the manuscript. All authors participated in the study.

## Conflict of Interest

The authors declare that the research was conducted in the absence of any commercial or financial relationships that could be construed as a potential conflict of interest.

## Publisher’s Note

All claims expressed in this article are solely those of the authors and do not necessarily represent those of their affiliated organizations, or those of the publisher, the editors and the reviewers. Any product that may be evaluated in this article, or claim that may be made by its manufacturer, is not guaranteed or endorsed by the publisher.

## Footnotes

1
https://pubmed.ncbi.nlm.nih.gov/

2
http://isiknowledge.com/wos

3
https://www.elsevier.com/zh-cn

4
https://www.cochranelibrary.com/

5
http://www.brainmap.org

6
http://rii.uthscsa.edu/mango/
